# Novel Fibroblast Growth Factor Receptor 3–Fatty Acid Synthase Gene Fusion in Recurrent Epithelioid Glioblastoma Linked to Aggressive Clinical Progression

**DOI:** 10.3390/curroncol31110539

**Published:** 2024-11-18

**Authors:** Miguel A. Diaz, Felisa Vázquez-Gómez, Irene Garrido, Francisco Arias, Julia Suarez, Ismael Buño, Álvaro Lassaletta

**Affiliations:** 1 Pediatric Hematology/Oncology, Hospital Infantil Universitario “Niño Jesús”, Universidad Autónoma de Madrid, 28009 Madrid, Spain; 2 Neuro-Radiology, Hospital General Universitario “Gregorio Marañón”, 28007 Madrid, Spain; 3 Pathology Department, Hospital General Universitario “Gregorio Marañón”, 28007 Madrid, Spain; farias@salud.madrid.org; 4 Genomics Unit, Hospital General Universitario “Gregorio Marañón”, 28007 Madrid, Spainismaelbuno@iisgm.es (I.B.); 5 Health Research Institute (IiSGM), Hospital General Universitario “Gregorio Marañón”, 28007 Madrid, Spain; 6 Department of Hematology, Hospital General Universitario “Gregorio Marañón”, 28007 Madrid, Spain; 7 Department of Cell Biology, School of Medicine, Universidad Complutense de Madrid, 28040 Madrid, Spain

**Keywords:** glioblastoma, FASN, FGFR3, fusion, epithelioid, fatty acid, radionecrosis, pseudoprogression

## Abstract

Glioblastoma (GBM) is the most common primary malignant brain tumor in adults, with a median overall survival (OS) of 15–18 months despite standard treatments. Approximately 8% of GBM cases exhibit genomic alterations in fibroblast growth factor receptors (FGFRs), particularly FGFR1 and FGFR3. Next-generation sequencing techniques have identified various FGFR3 fusions in GBM. This report presents a novel FGFR3 fusion with fatty acid synthase (FASN) in a 41-year-old male diagnosed with GBM. The patient presented with a persistent headache, and imaging revealed a right frontal lobe lesion. Surgical resection and subsequent histopathology confirmed GBM. Initial NGS analysis showed no mutations in the IDH1, IDH2 or H3F3 genes, but revealed a TERT promoter mutation and CDKN2A/2B and PTEN deletions. Postoperative treatment included radiotherapy and temozolomide. Despite initial management, recurrence occurred four months post-diagnosis, confirmed by MRI and histology. A second surgery identified a novel FGFR3-FASN fusion, alongside increased Ki67 expression. The recurrence was managed with regorafenib and bevacizumab, though complications like hand–foot syndrome and radiation necrosis arose. Despite initial improvement, the patient died 15 months after diagnosis. This case underscores the importance of understanding GBM’s molecular landscape for effective treatment strategies. The novel FGFR3-FASN fusion suggests potential implications for GBM recurrence and lipid metabolism. Further studies are warranted to explore FGFR3-FASN’s role in GBM and its therapeutic targeting.

## 1. Introduction

Glioblastoma (GBM) is the most frequent primary malignant brain tumor in adults [1]. Current treatment options include surgical resection, radiotherapy (RT) and concomitant and/or adjuvant chemotherapy [2]. However, GBM is a fatal disease with a median overall survival (OS) of 15 to 18 months in well-selected patients after standard care, and the 5-year OS is less than 10% [2,3,4,5]. GBM is categorized into distinct molecular subtypes, some of which influence the response to therapeutic interventions [4,6,7,8,9,10].

Fibroblastic growth factor receptors (FGFRs) belong to the family of tyrosine kinase (TK) receptors and play a crucial role in the development and progression of various cancers [11]. FGFR genomic alterations including amplification, mutations and fusions are observed in approximately 8% of GBMs, with majority of aberrations occurring in the FGFR1 and FGFR3 genes [12]. Next-generation sequencing (NGS) techniques have emerged as a pivotal method for identifying FGFR gene fusions in clinical samples [13]. The predominant fusion partner for FGFR3 in GBM patients is the transforming acidic coiled-coil 3 (TACC3), resulting in an in-frame fusion of the FGFR3 N-terminus with the TACC3 C-terminus. Other fusion partners of FGFR3 have been reported in various tumors including BAIAP2L1, AES, ELAVL3, JAKMIP1, TNIP2 and WHSC1 [11,12,13].

We present the identification, utilizing NGS techniques, of a previously unreported FGFR3 fusion with fatty acid synthase (FASN) in a patient diagnosed with GBM. To our knowledge, this is the first documented instance of an FGFR3-FASN fusion in the context of GBM. 

## 2. Case Report

**Figure 1:**
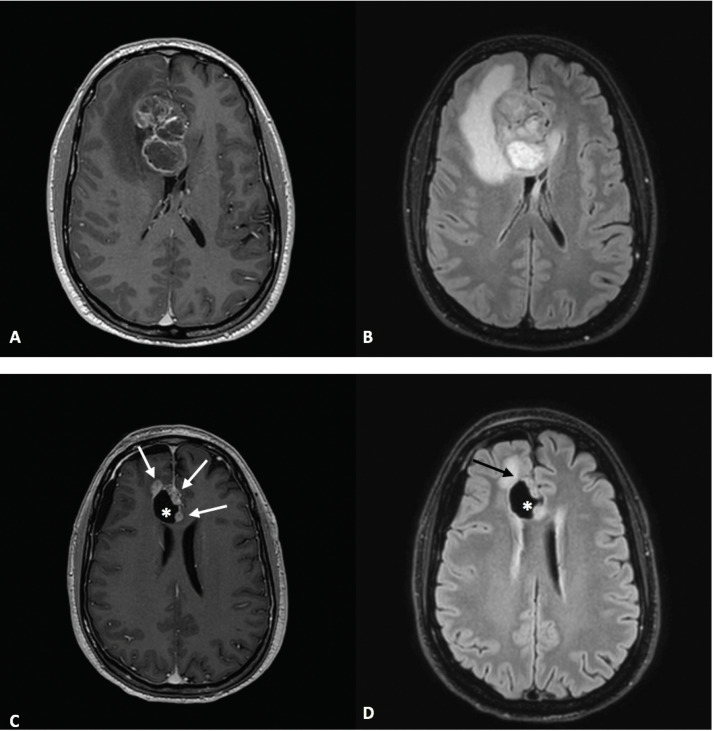
MRI at time of diagnosis and progression. Diagnosis: Axial T1 contrast-enhanced image (**A**) shows a right parasagittal frontal tumor involving the genu of the corpus callosum. There is predominantly peripheral mural enhancement with central areas of necrotic appearance. It is surrounded by a diffuse hyperintense area on the FLAIR sequence (**B**), mostly due to edema. Progression: Five months after diagnosis, several enhancing nodules (white arrows in (**C**)) appear along the anterior and medial margins of the post-surgical cavity (*), suggestive of tumor recurrence. There is scarce edema in the anterior frontal subcortical white matter on the FLAIR image (black arrow in (**D**)). * refers to post-surgical cavity.

In late August 2022, a 41-year-old male presented to the emergency department with a 5-day history of a persistent headache. He denied experiencing any other significant neurological or systemic symptoms. Upon physical examination, no focal neurological deficits were noted, and the patient exhibit full strength in all extremities without sensory impairments. The patient’s Karnofsky Performance Score (KPS) was recorded as 90%. Gadolinium-enhanced brain magnetic resonance imaging (MRI) revealed a heterogeneously expansive lesion measuring 49 × 35 × 43 mm in the right frontal lobe, accompanied by diffuse perilesional edema affecting the white matter and resulting in midline shift (Figure 1). 

During surgery, intraoperative pathology indicated the presence of a primary glial neoplasm. The patient underwent maximal safe resection. Microscopic examination of hematoxylin-and-eosin-stained sections revealed an epithelioid neoplasia primarily distributed in the perivascular areas. The neoplastic cells exhibited epithelioid morphology, marked pleomorphism, a large cytoplasm, numerous atypical mitoses and moderate eosinophilia. In certain regions, tumor cells demonstrated an astrocytic phenotype within a disorganized fibrillar stroma. Microvascular proliferation, hemorrhage, necrosis and microcalcifications were also observed. An infiltrative interphase with adjacent brain parenchyma was noted. 

Malignant cells exhibited diffuse reactivity for glial fibrillary acid protein (GFAP) but showed no reactivity for melanoma-associated antigens or rare epithelial metastatic tumors such as Melan-A, HMB45 or CAM5.2. Ki67 expression reached 15% in some tumor areas, while p53 expression was <10% in tumor cells. Following DNA and RNA extraction and purification, NGS analysis (MiSeq^TM^, Illumina Inc.; San Diego, CA, USA) revealed the absence of mutations in the isocitrate dehydrogenase (IDH) 1, IDH2 and H3F3 genes. TERT promoter mutation (chr 5:1.295.250>T) (C250T) with a variant allelic frequency of 32.7% was detected. No other genomic alterations, including BRAF-V600E/E2/D and V600K/R/M mutations, were observed. Multiplex ligation probe amplification (MLPA) analysis revealed CDKN2A/2B and PTEN biallelic deletions. A methylation-specific MLPA study for the O^6^-methylguanine-DNA methyltransferase (MGMT) gene indicated 15% methylation (unmethylated sample). According to the 2021 WHO classification, a diagnosis of grade 4 glioblastoma IDH-wildtype and H3-wild type was delivered. 

Following a 48 h postoperative brain MRI indicating no residual disease, the patient was discharged with no complications. Five weeks later, he commenced RT delivering 60 Gy in 30 fractions over six weeks, concurrent with TMZ at a dose of 75 mg/m^2^ for 6 to 7 weeks, followed by 150 mg/m^2^ per day for 5 days every 28 days for up to six cycles beginning one month after RT. The patient experienced headaches and fatigue somnolence syndrome during RT, managed with dexamethasone without treatment interruption.

**Figure 2:**
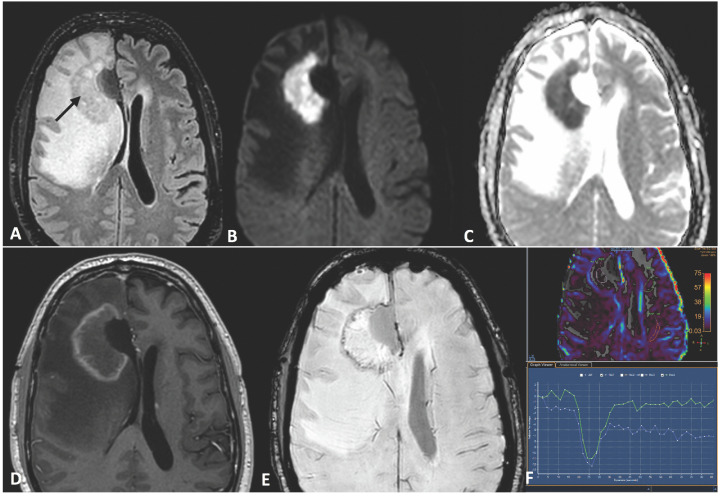
Urgent MRI in May 2023, showing an intermediate signal lesion on FLAIR imaging (black arrow in (**A**)) adjacent to the lateral edge of the resection cavity, with increased edema and a worsening mass effect, causing midline deviation. There is a markedly high signal intensity on diffusion-weighted imaging (DWI) (**B**), with very low apparent diffusion coefficient (ADC) values due to diffusion restriction (**C**), mild peripheral enhancement on the T1 CE sequence (**D**) and an irregular surrounding low signal (hemorrhage and hemoglobin degradation products) with very fine lineal hypointensities within the lesion (probably vascular structures) on susceptibility-weighted imaging (SWI) (**E**). On dynamic susceptibility contrast (DSC) perfusion imaging (**F**), relative cerebral blood volume is slightly increased (blue dynamic curve in (**F**)) with respect to the normal contralateral white matter (green dynamic curve in (**F**)).

**Figure 3:**
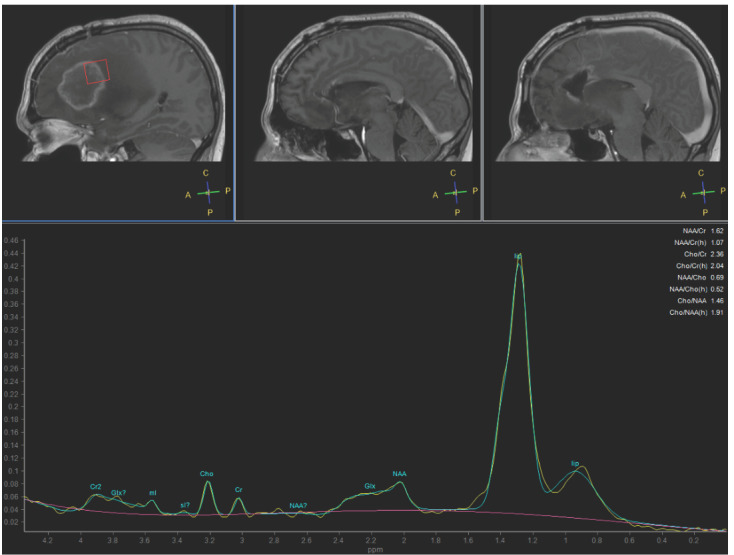
Short-echo-time MR spectroscopy shows a mildly elevated choline/creatine (Cho/Cr) ratio, low N-acetylaspartate/creatine ratio and a very high peak of lipids. Radionecrosis was suspected, but a differential diagnosis with pseudoprogression was considered.

**Figure 4:**
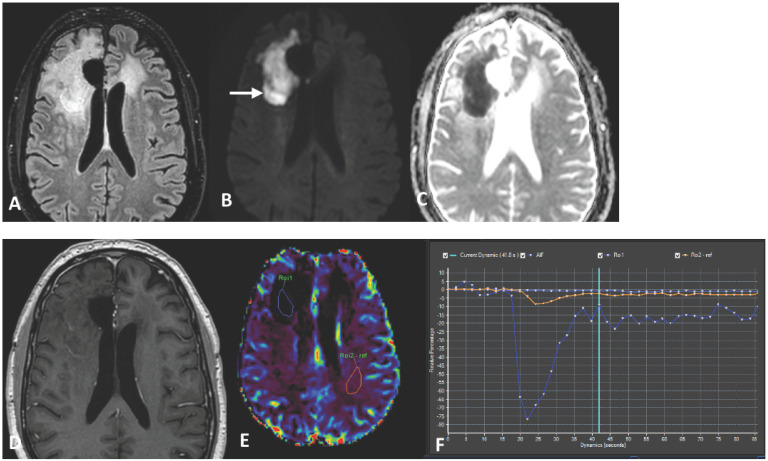
The last MRI, performed fifteen months after diagnosis, shows less edema and an improvement in the mass effect (**A**). Marked DWI restriction persists (**B**) with very low ADC values (**C**). There is a slight growth in the lesion from its posterior margin (white arrow in (**B**)). Peripheral enhancement has decreased on the T1 CE sequence (**D**). DSC perfusion imaging shows decreased rVSC compared to the normal contralateral white matter (**E**), with an almost flat dynamic curve (light blue in (**F**)). Persistent marked DWI restriction, but contrast enhancement improvement, and decreased perfusion suggest a mixture of mechanisms, partly radionecrosis with some degree of superimposed pseudoprogression, due to regorafenib.

**Figure 5:**
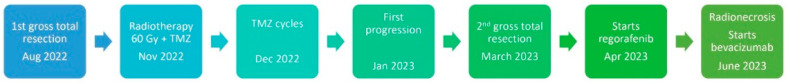
Timeline describing the treatment of the patient.

In January 2023, MRI revealed contrast enhancement in the surgical bed (Figure 1). Twenty days later, an amino acid tracer positron emission tomography (PET) scan with ^18^Fluoro-L-dihydroxy-phenyalanine (F-DOPA) showed increased tracer incorporation, prompting a programmed second surgery in March 2023, confirming glioblastoma recurrence. Histologic features included typical glioblastoma morphology with endothelial proliferation and tumoral necrosis. Additionally, evident blood vessel damage indicative of radiation necrosis was observed. Ki67 expression increased to 25%. NGS analysis identified a TERT promoter mutation (chr 5:1.295.250>T) (C250T) with a variant allelic frequency of 25% and a novel FGFR3-FASN gene fusion (chr4:1808678; 17-FGFR3 and chr17:80049544; 9-FASN). No other genomic alterations were detected. Since there is no consensus on treatment for recurrent GBM, the patient was included in a compassionate use program of regorafenib for recurrent GBM. Second-line treatment with regorafenib was initiated at 160 mg/day for 21 days of a 28-day cycle but reduced to 120 mg/day due to severe hand–foot syndrome toxicity. During the second regorafenib cycle in May 2023, the patient experienced high-grade fever followed by a progressive worsening of headaches, left facial paresis, somnolence and fatigue. An urgent MRI study showed an increased size of a contrast-enhancing lesion near the irradiated tumor volume, affecting the periventricular white matter, and a significant increase in perilesional edema with a shift of the left lateral ventricle. The contrast-enhancing lesion showed a low relative cerebral blood volume (rCBV). MR spectroscopy showed low ratios of choline/creatine (Cho/Cr) and choline/N- acetylaspartate (Cho/NAA) and a high lipid peak (Figure 2 and Figure 3). A clinical diagnosis of radionecrosis was established. High-dose steroids were administered with rapid clinical improvement, followed by intravenous bevacizumab at a dose of 7.5 mg/kg every 15 days and then every 21 days, resulting in progressive reduction in the steroid dosage and disappearance of the cerebral edema on MRI studies (Figure 4). Despite dermatologic toxicity from regorafenib, which led to a dose reduction at 80 mg/day and eventually to 40 mg/day, the patient maintained a KPS > 80%. Eight months after starting regorafenib treatment and six months following bevacizumab, the patient died suddenly, presenting a decorticate posture. Resuscitation attempts were unsuccessful and a postmortem study was not performed. The patient survived 15 months from diagnosis (Figure 5).

## 3. Discussion

Understanding the molecular composition of post-treatment residual GBM cells is pivotal for devising effective therapies. However, the precise mechanisms driving recurrence remain poorly understood. Roughly speaking, two recurrence models have been proposed after the genomic comparison of primary tumors and their recurrences [14]. In the ancestral cell origin model, treatment may remove all dominant disease clones but not refractory ancestral cells, which accumulate new mutations. Mutations shared by primary and recurrent tumors are presumably derived solely from the ancestral cells, leading to increased divergence between subsequent tumors. In the clonal evolution model, treatment removed most of the primary tumor cells, but select resistant clones formed from the major primary disease clones. Additional mutations were accumulated and therefore detected in the recurrent tumor, and all primary clonal mutations were retained at recurrence. However, as only a small percentage of patients at the time of clinical and/or radiological recurrences undergo histologic confirmation, the models rely on data from only a few studies comparing genomic changes in matched pairs of primary and relapsed tumors [14]. Korber et al. [15] analyzed 21 paired primary and locally relapsed IDH-wild-type GBMs and showed that most GBMs are initiated by gains and losses of specific chromosomes. The inferred evolutionary trajectories of primary/relapsed GBM pairs indicate that TERT promoter mutations often occur later as a prerequisite for rapid growth, and relapsed GBMs acquire few stereotypical mutations.

Epithelioid glioblastoma (EG) is a rare and aggressive variant of IDH-wild type GBM, which often coexists with other types of diffuse glioma [16,17]. Approximately half of EG cases carry a BRAF V600E mutation [16]. EG may exhibit greater genetic instability and an elevated number of mutations compared to typical glioblastomas [18]. In the present case, histopathology analysis revealed distinct regions characterized by atypical epithelioid morphologies alongside typical glioblastoma features. Immunohistochemistry demonstrated GFAP positivity but was negative for IDH-1 and p53, indicating the wild type for both markers. A molecular study showed that the promoter region of the DNA repair enzyme MGMT was unmethylated. Thus, the findings were consistent with a grade 4 IDH-1 wild-type glioblastoma [9]. In a recently published case report of GBM with glial and epithelioid components, the genomic and transcriptomic profiles showed that both glioma and epithelioid sections contained identical point mutations in PTEN, RB1, TERT promoter and TP53. Electronic karyotype analysis also revealed similar chromosomal copy number alterations, but the epithelioid component showed additional abnormalities that were not found in the conventional glioblastoma component [18]. Recently, a case of clonal evolution during the treatment of EG has been reported [19]. Compared to the initial resected tumor specimen, the recurrent tumor possessed a loss of heterozygosity of the 1p, 10q, 17q and 19q chromosomes, as well as a new C228T TERT promoter mutation, while the status of wild-type IDH1/IDH2 was unchanged. These data suggest that EG may exhibit increased genetic instability, making it prone to the development of additional genetic mutations, copy number alterations or both over time [19]. 

In the present case, tumor recurrence occurred 4 months after diagnosis, based on MRI findings and confirmed by histology after the second surgery. Recurrence analysis revealed molecular findings not previously identified, including a novel FGFR3-FASN gene fusion added to the previous molecular profile.

Deregulation of FGFR signaling plays a significant role in the development and progression of several cancers [11]. FGFR3 fusions are most frequently observed in GBM, lung and bladder cancers [11,13]. Most FGFR3 fusions are with TACC3 as the gene partner and have been described in several types of cancers including GBM [11,13,20,21,22]. FGFR3-TACC3 fusion protein induces a constitutive activation of the TK domain [23]. Preclinical studies demonstrated that the presence of FGFR3-TACC3 fusion increased cancer cell lines’ proliferation and tumorigenesis in mice [21]. FGFR3 fusions have been described with other partners [13], but to the best of our knowledge, FGFR3-FASN fusion has not been previously described [24]. 

FASN is physiologically expressed in liver cells and lipogenic tissue [25]. Its overexpression is driven at a transcriptional level by growth factor stimulation, oncogene activation or tumor suppressor loss. In GBM, lipid metabolism is altered to utilize fatty acid for sustained cellular growth [26,27,28,29,30,31]. Two key enzymes, acetyl-CoA carboxylase (ACC) and FASN, mediate fatty acids’ biosynthesis. FASN catalyzes the “de novo” synthesis of fatty acids and is deregulated in several cancers, including GBM [30,31]. Both ACC and FASN are regulated by SREBP-1, which responds to the EGFR-PI3K-Akt1 signaling pathway promoting GBM cells’ proliferation through the synthesis of polyunsaturated fatty acids [32]. Additionally, in marginal and hypoxic regions, fatty acids are stored and form structures with polyunsaturated fatty acids known as pseudo-palisades [30,31]. 

FASN overexpression has been described in human glioma cell lines and human glioma tissue samples, as compared to the normal human brain [29,30]. More recently, overexpression of FASN in gliomas has been correlated with the WHO grades [29]. Moreover, a recent paper by De Martino et al. showed that “in vitro” and “in vivo” irradiated GBM tumors switch their metabolic program to accumulate lipids, especially unsaturated fatty acids [31]. These results suggest that RT drives GBM resistance by generating a lipogenic environment permissive to GBM survival. FASN can be used as a biomarker since it is enriched in extracellular vesicles derived from GBM cell lines and human plasma, suggesting that plasma extracellular vesicle levels could be used as a noninvasive biomarker for GBM [27]. 

We can only speculate regarding the role, if any, of FGFR3-FASN fusion in the recurrence observed in our case. However, it is noteworthy that this gene fusion was not detected at the time of diagnosis. An early recurrence occurred 3 months after the completion of radiotherapy, which might be associated with the newly described gene fusion or, alternatively, with the patient’s condition of unmethylated MGMT, a well-known risk factor associated with a shorter overall survival [9,17]. The increase in Ki67 expression from an initial 15% to 25% at recurrence is noteworthy. High Ki67 expression has been associated with a shorter OS in GBM patients [17,27].

As there is no widely accepted treatment for GBM recurrence, our case highlights various treatment options that warrant consideration [33]. Despite the controversial impact on OS, we opted for a second surgery approach based on factors that supported the possibility of a successful subsequent surgery, such as the maximal safe resection, the frontal right lobe location, the patient’s good clinical condition with no focal neurological sequelae, and the absence of associated comorbidities [34]. Additionally, a second surgery might provide new histological and molecular findings that could guide the treatment going forward. 

Once tumor progression was confirmed, continuing temozolomide was deemed futile. TK inhibitors like erdafitinib [35] and infigratinib [36] could be useful for rescue treatment of FGFR-altered recurrent gliomas. However, currently, there is neither an accepted indication nor a compassionate use program for those TKIs in the treatment of recurrent GBM.

Regorafenib is an oral multi-kinase inhibitor against several targets such as VEGFR1-3, TIE2, KIT, RET, RAF1, BRAF, PDGFR and FGFR. It is approved as a monotherapy for treating hepatocellular carcinoma, gastrointestinal stromal tumors and colorectal cancer [37]. The REGOMA trial, which tested regorafenib in recurrent GBM patients, showed a significant improvement in OS in the regorafenib arm when compared with the lomustine arm [38]. However, a consensus regarding the indication of regorafenib for the treatment of recurrence remains elusive. We elected to administer regorafenib in our case based on published results [39] and the tangible prospect of accessing the drug promptly. 

In GBM, assessing the response during the follow-up after treatment poses challenges, particularly in distinguishing treatment-related changes on MRI from tumor progression, especially with the introduction of new drugs like regorafenib [40,41]. Radiation necrosis (RN) represents an infrequent yet potentially severe late complication of RT, resulting from significant co-irradiation of brain tissue [42]. RN typically occurs 3–12 months post-radiotherapy though it can also manifest years or even decades later [43]. Notably, a higher incidence of RN has been observed in patients who underwent surgery for progressive brain lesions within the first 6 months following combined chemoradiotherapy with temozolomide, as seen in our case [44]. In adults, the reported incidence of RN following radiotherapy for brain tumors ranges from 3% to 24%. The clinical course of RN varies widely [43,44]. While many cases of RN remain asymptomatic, a substantial portion may present with clinical symptoms [40]. In severe cases, clinical progression can be fatal. Most lesions consist of a contrast-enhancing mass on T1-weighted imaging with gadolinium, making them indistinguishable from tumor progression with conventional MR techniques. On T2-weighted images, the solid portion of the radiation-induced necrotic mass shows low signal intensity, while the central necrotic component shows increased signal intensity [42]. MR spectroscopy can differentiate residual or recurrent tumors from pure treatment-related necrosis, but is less effective in distinguish mixed necrosis and tumor tissue. Diffusion-weighted imaging (DWI) has also been evaluated for differentiating tumor from necrosis post-radiotherapy. The apparent diffusion coefficient was noted to be higher in necrotic tissue than in recurrent tumor [45]. RN often occurs in the periventricular white matter, likely due to the poor blood supply from long medullary arteries, which lack collateral vessels, making this area vulnerable to the ischemic effects of radiation vasculopathy. The underlying mechanisms of RN are not fully understood. However, hypoxia-induced factor 1-alpha (HIF-1A) in thought to play a role in initiating RN by overproducing VEGF, leading to cerebral edema [46]. Steroids are generally effective in controlling edema, though their long-term use can result in significant side effects. Recently, bevacizumab has shown effectiveness in patients with malignant glioma diagnosed with RN via MRI and biopsy. The symptomatic control obtained in these patients supports the notion of a role for VEGF signaling pathways in treatment-related necrosis [47]. In our case, the concurrent administration of bevacizumab and regorafenib resulted in a swift alleviation of symptoms linked to cerebral edema and facilitated the gradual reduction and eventually cessation of steroid therapy. However, our treatment approach was impeded by drug toxicity, notably severe hand and foot syndrome, necessitating a substantial reduction in drug dosage to enhance treatment tolerability [39]. 

Determining the cause of death (COD) in GBM patients poses considerable challenges, primarily due to its multifactorial nature [48]. While clinical tumor progression typically precedes death in most cases, this was not the case in our patient. Remarkably, the patient increased the interval of bevacizumab administration to 3 weeks and ceased steroid treatment a month prior to his death, experiencing a discernible improvement in his overall life conditions. The sudden event causing his death in this case points to cerebral hemorrhage as the COD, likely due to radiotherapy vascular cerebral damage and/or bevacizumab toxicity. Cranial RT has been associated with several late effects on the cerebrovascular system including lacunar infarcts, vascular malformations and hemorrhage. Likewise, bevacizumab treatment has a low but not negligible risk of intracranial hemorrhage [49,50]. Moreover, given the infiltrating nature of GBM, the tumor itself could not be ruled out as the COD [51]. Brain postmortem examination remains the definitive method not only for assessing the effects of treatment and the topography of tumor infiltration but also for determining the COD and acquiring novel genetic and molecular insights at the time of death. 

In conclusion, the identification of the previously unreported FGFR3-FASN gene fusion offers novel insights into molecular events potentially linked to early recurrence in GBM and highlights the potential involvement of lipid metabolism in GBM biology. 

## Data Availability

The original contributions presented in this study are included in the article, and further inquiries can be directed to the corresponding author.
